# Comparison of conventional medicine, TCM treatment, and combination of both conventional medicine and TCM treatment for patients with chronic obstructive pulmonary disease: study protocol of a randomized comparative effectiveness research trial

**DOI:** 10.1186/1745-6215-15-153

**Published:** 2014-05-01

**Authors:** Jian-sheng Li, Yang Xie, Su-yun Li, Xue-qing Yu

**Affiliations:** 1 The Geriatric Department, Henan University of Traditional Chinese Medicine, Longzihu University Town, Zhengdong New District, Zhengzhou, People's Republic of China; 2 Department of Respiratory, The First Affiliated Hospital of Henan University of Traditional Chinese Medicine, Renmin Road 19, Zhengzhou, People's Republic of China; 3 Collaborative Innovation Center for Respiratory Disease Diagnosis and Treatment & Chinese Medicine Development of Henan Province, Zhengzhou, People's Republic of China

**Keywords:** Chronic obstructive pulmonary disease, Comparative effectiveness research, Traditional Chinese medicine

## Abstract

**Background:**

Chronic obstructive pulmonary disease (COPD) affects millions worldwide. Although many therapies exist and are being developed to relieve symptoms and reduce mortality, few data are available to understand which of the therapeutic alternatives is the most cost-effective for COPD patients in everyday clinical practice, especially for traditional Chinese medicine (TCM). Comparative effectiveness research can help patients, clinicians, and decision-makers make best informed treatment decisions where such evidence was previously lacking. This study aims to compare the effectiveness and economic evaluation of three treatments: (1) conventional Western medicine; (2) TCM treatments, which have been evaluated and have certain effect; and (3) a combination of both conventional Western medicine and TCM treatments, and then determine which treatment is the most suitable for COPD patients.

**Methods/design:**

A multicenter, pragmatic, randomized, controlled trial is adopted. A total of 360 patients will be recruited and randomly assigned to one of the three treatments group, with 120 in each group. Patients in the conventional Western medicine group will be given Salbutamol, Formoterol, Salmeterol/fluticasone, respectively, according to the guidelines. For the TCM group, patients will be given Bufei granule, Bu-Fei Jian-Pi granule, Bu-Fei Yi-Shen granule, and Yi-Qi Zi-Shen granule based on their corresponding TCM syndrome patterns, respectively. For the combination of conventional medicine and TCM treatments group, patients will be given a combination of conventional Western medicine and TCM granules. Treatments in each group are recognized as a whole comprehensive intervention. After the 26-week treatment, another 26 weeks will be followed up. The outcome measures including the frequency and duration of acute exacerbations, lung function, dyspnea, exercise capacity, quality of life, and economic evaluation will be assessed.

**Discussion:**

It is hypothesized that each of the three treatments will have beneficial effects in reducing the frequency and duration of acute exacerbations, improving exercise capacity and psychosocial function of COPD patients. In addition, the combination of conventional medicine and TCM treatments may be most suitable for COPD patients with better effectiveness and economic evaluation.

**Trial registration:**

ClinicalTrials.gov NCT01836016.

## Background

Chronic obstructive pulmonary disease (COPD) is a common, preventable, and treatable disease characterized by progressive airflow limitation. COPD affects millions worldwide and remains a leading cause of morbidity, mortality, and elevated healthcare costs worldwide [1]. The projection for 2030 indicates that COPD will be the third leading cause of death worldwide [2]. In China, the overall prevalence is 8.2% in individuals aged 40 years or older, and an estimated 65 million people will die of COPD between 2003 and 2033 [3]. In the United States, the total economic cost of COPD was $49.9 billion in 2010, which included approximately $29.5 billion in direct expenditures per year [4]. Hence, strategies and management for both primary and secondary prevention and treatment should be well developed.

Although many therapies exist and are being developed to relieve symptoms and reduce the mortality, most were demonstrated in placebo-controlled efficacy studies with highly selected patient populations in tightly controlled study settings [5,6]. Few data are available to compare the therapeutic alternatives by randomized clinical trials in everyday clinical practice. Much of the comparative research evidence for COPD was obtained by observational studies [7]. Furthermore, studies in real world populations of COPD who received different therapies that do have efficacy evidence were found wide variations in care delivery [8]. The above considerations highlight the need to identify the most cost-effective therapies and translate them into healthcare for millions of COPD patients [9]. In short, there is a need for comparative effectiveness research (CER) in COPD.

CER has received growing attention worldwide [10], which is defined as ‘the conduct and synthesis of research comparing the benefits and harms of different interventions and strategies to prevent, diagnose, treat, and monitor health conditions in “real world” settings’ [11]. The direct comparisons of treatments provided by CER can help patients, clinicians, and decision-makers make best informed treatment decisions where such evidence was previously lacking [12], especially for traditional Chinese medicine (TCM).

The remarkable longevity and current popularity of TCM for COPD implies its potential advantages. Our previous randomized controlled trials on comprehensive TCM interventions, especially based on the TCM patterns, have certain evidence indicating definite effect for COPD patients [13,14]. When facing many treatment approaches, it is difficult to identify the most suitable treatment. Therefore, this study aims to compare the effectiveness and economic evaluation of three treatments: (1) conventional Western medicine; (2) TCM treatments, which have been evaluated and have certain effect; and (3) a combination of both conventional Western medicine and TCM treatments, and then determine which treatment is the most suitable for COPD patients.

## Methods/design

### Study design

This is a multicenter, pragmatic, randomized, controlled trial to evaluate the effectiveness and economic evaluation of three treatments in COPD patients. A total of 360 patients will be recruited and randomly assigned to one of the three treatments group. After the 26-week treatment period, another 26 weeks will be followed up. The outcome measures including the frequency of exacerbations, lung function, dyspnea, exercise capacity, quality of life, and economic evaluation will be assessed.

### Participants

#### Inclusion criteria

To participate in the study, COPD patients should meet the following criteria: (1) the diagnostic criteria of COPD [15,16]; (2) the TCM syndrome pattern criteria [14,17] (pattern of lung qi deficiency, pattern of lung-spleen qi deficiency, pattern of lung-kidney qi deficiency, pattern of lung-kidney qi and yin deficiency); (3) medically stable and confirmed diagnosis of mild to very severe COPD; (4) aged between 18 and 80 years; (5) without participations in other interventional trials in the previous 1 month; (6) with informed signed consent.

#### Exclusion criteria

Patients will be excluded for the following reasons: (1) pregnant or breast-feeding women; (2) any psychiatric condition rendering the patient unable to understand the nature, scope, and possible consequences of the study; (3) malignancy for which patient has undergone resection, radiation therapy, or chemotherapy within the last 5 years; (4) current respiratory disorders other than COPD (for example, bronchiectasis, tuberculosis, lung fibrosis, pulmonary thromboembolic); (5) complicated with heart failure (Grade III or IV New York Heart Association Functional Classification) or myocardial infarction within 6 months or unstable hemodynamics; (6) complicated with serious hepatic and renal diseases (liver cirrhosis, portal hypertension, bleeding of varicose veins, dialysis, or renal transplantation); (7) participating in other trials or allergic to the used medicine.

### Ethics and recruitment

All patients will sign the informed consent before inclusion. The study has been approved by the Ethical Research Committees of the First Affiliated Hospital of Henan University of Traditional Chinese Medicine with identifier 2013HL004-01. Any revisions of the study protocol will be submitted to the ethics committee.

COPD patients will be recruited from either the outpatient department or open recruitment and be observed in six research centers, which are the First Affiliated Hospital of Henan University of Traditional Chinese Medicine, Shuguang Hospital Affiliated to Shanghai University of Traditional Chinese Medicine, The Second Affiliated Hospital of Liaoning University of Traditional Chinese Medicine, Shanxi Provincial Traditional Chinese Medicine, The Affiliated Hospital of Jiangxi University of Traditional Chinese Medicine, and Henan University Huaihe Hospital. Recruitment will last for a period of 6 months from November 2013 or until a sample of 360 patients are enrolled.

### Sample size

The frequency of acute exacerbation of COPD is considered as the primary outcome. The formulae ( $n=\psi^{2}\left(\sum S_{i}^{2}/k\right)/\left[\sum {\left({\bar{X}}_{i}-\bar{X}\right)}^{2}/\left(k-1\right)\right]$) was based on a comparison among the equal numbers of a multiple samples mean. From a previous study [13,14], the number of the exacerbation frequency was 1.17 times every year by treatment of conventional medicine, and that was 0.97 times every year by treatment of TCM, and that was 0.68 times every year by treatment of both conventional medicine and TCM. The two-sided alpha level was 0.05, and the beta level was 0.01, the ψ value was 2.52, the k value was 3. Using the calculation, the sample size in each group was approximately 100. Allowing for a 20% dropout rate over the course of the study, the sample size would be approximately 120.Therefore, the total sample size was 360, with 120 in each group.

### Randomization

Treatment allocation will occur when the participants meet the inclusion criteria and sign the informed consent form. A stratified and block randomization design is adopted. The number of the groups is three and the distribution ratio is 1-to-1. Considering the long time for treatment observation, the process is divided into more than one block, and the length of the block is six. The number of center hierarchical levels was six. A random number from 001 to 360 is generated by SAS 9.2 and saved in a sealed envelope by an independent clinical statistician. In the event of a clinical emergency, the individual’s randomization code and group allocation could be identified by the emergency envelope as soon as possible. The randomization design is provided by the DME department of Nanjing University of Traditional Chinese Medicine.

### Interventions

Patients in the conventional medicine group will be divided into Groups A, B, C, or D, and then given Salbutamol, Formoterol, Salmeterol/fluticasone for 26 weeks, respectively, according to the Global Initiative for Chronic Obstructive Lung Disease (GOLD) and Chinese Treatment Guidelines. The choice within each group of medication commonly used in treating COPD depends on the patient-specific combined COPD assessment as the relationship between severity of symptoms, risk of exacerbations, drug availability, and the patient’s response. The pharmacologic therapy (three drugs) for COPD patients is recognized as a whole comprehensive intervention rather than one drug for each group. The whole comprehensive Western intervention is shown in Table 1.

**Table 1:** Comprehensive Western intervention for stable COPD patients

**Classification**	**Therapy**
Group A	Salbutamol (Ventolin®, GlaxoSmithKline) 100 μg/dose, 200 inhalations. Dosing: one inhalation of 100 μg each time (when needed), and the maximum dose is 8 to 12 inhalations a day
Group B	Formoterol (Oxis Turbuhaler®, AstraZeneca), 4.5 μg/dose, 60 inhalations. Dosing: one inhalation of 4.5 μg each time, twice daily
Group C	Salmeterol/fluticasone (Seretide®, GlaxoSmithKline), 50/500 μg/dose, 60 inhalations. Dosing: one inhalation of 50/500 μg each time, twice daily
Group D	Salmeterol/fluticasone (Seretide®, GlaxoSmithKline), 50/500 μg/dose, 60 inhalations. Dosing: one inhalation of 50/500 μg each time, twice daily

For the TCM treatments group, patients will be given herbal interventions based on the TCM syndrome patterns, respectively, which are Bufei granule for lung qi deficiency, Bu-Fei Jian-Pi granule for lung-spleen qi deficiency, Bu-Fei Yi-Shen granule for lung-kidney qi deficiency, and Yi-Qi Zi-Shen granule for lung-kidney qi and yin deficiency. The herbal interventions are also recognized as a whole comprehensive intervention. The TCM granules are compound preparations of Chinese herbs and its main components are shown in Table 2. Each type of granule comes in packs of four bags. Each bag of Bu-Fei granule (batch number: 1305402) contains 6.35 g, and that of Bu-Fei Jian-Pi granule (batch number: 1305403) is 5.82 g, Bu-Fei Yi-Shen granule (batch number: 1305404) is 5.33 g, Yi-Qi Zi-Shen granule (batch number: 1305405) is 5.37 g. These components of the TCM granules are produced and packed by Jiang Yin Tian Jiang Pharmaceutical Co. Ltd. with the authentication quality of Goods Manufacturing Practice (Approval Number: SU J0677), Jiangsu, PR China. The test results of drug quality were consistent with the required quality standards. Each type of granule will be given orally, two bags each time, twice a day for 26 weeks.

**Table 2:** Main components of traditional Chinese medicine treatment

**Chinese name**	**Latin name**	**Amount (g)**
**Bu**-**Fei granule for pattern of lung qi deficiency**		
Dang Shen	Codonopsis pilosula	15
Ai Di Cha	*Herba ardisiae Japonicae*	15
Zi Wan	*Radix asteris*	9
Chen Pi	*Pericarpium citri reticulatae*	9
**Bu**-**Fei Jian**-**Pi granule for pattern of lung**-**spleen qi deficiency**		
Huang Qi	*Astragalus propinquus*	15
Dang Shen	*Codonopsis pilosula*	15
Bai Zhu	*Atractylodes macrocephala*	12
Fu Ling	*Wolfiporia extensa*	12
**Bu**-**Fei Yi**-**Shen granule for pattern of lung**-**kidney qi deficiency**		
Ren Shen	*Radix Ginseng*	9
Huang Qi	*Astragalus propinquus*	15
Gou Qi Zi	*Lycium barbarum*	12
Shan Zhu Yu	*Cornus officinalis*	12
Yin Yang Huo	*Epimedium brevicornu*	9
**Yi**-**Qi Zi**-**Shen granule for pattern of lung**-**kidney qi and yin deficiency**		
Ren Shen	*Radix Ginseng*	9
Huang Jing	*Polygonatum sibiricum*	15
Shu Di Huang	*Rehmannia glutinosa*	15
Mai Dong	*Ophiopogon japonicus*	15
Wu Wei Zi	*Schisandra chinensis*	9

For the combination of conventional medicine and TCM treatments group, patients will be given the combination of conventional Western medicine and TCM granules as stated above for 26 weeks.

### Outcome measure

#### Primary outcome measure

##### Exacerbations of COPD

The frequency and duration of acute exacerbations of COPD (AECOPD) is the primary outcome measure. AECOPD is characterized by a change in patients’ original conditions of dyspnea, cough, and (or) expectoration in the development of the disease, which is beyond daily routine variation, and requires a change in regular medication [18]. Its reductions are a major goal of COPD management and an important indicator for evaluating the treatments. The frequency of AECOPD is the interval between two acute exacerbations in more than 1 week. If the interval between two onsets of acute exacerbation is within 1 week, it is counted as one number acute exacerbation. The duration of AECOPD is measured from the beginning of the acute exacerbation to the symptoms the patient feels reduce significantly or restore to the level before acute exacerbation. The number of frequency and duration of AECOPD occurred each time during the 26-week treatment period and 26-week follow-up, and then count the total number and average number of frequency and duration.

#### The secondary outcome measures

##### Lung function

The indicators of forced vital capacity (FVC), forced expiratory volume in 1 s (FEV1) and FEV1 percentage of the predicted value (FEV1%) will be tested. A positive change from baseline in them will indicate the improvement in lung function.

##### Dyspnea

The Modified Medical Research Council (MMRC) by the American Thoracic Society [19] will be assessed to evaluate the level of dyspnea. The MMRC scale is a simple grading system that scored from 0 (less severe) to 4 (severe).

##### The 6-minute walking distance (6MWD)

This is to evaluate the distance a person can walk on a flat surface in 6 min to assess the exercise capacity.

##### Quality of life

The COPD Assessment Test (CAT) will be adopted. The CAT is the self-complete questionnaires with eight items, each formatted as a semantic six-point ranging from 0 to 5. Range of CAT scores from 0 to 40. Higher scores denote a more severe impact of patient’s quality of life [20]. The patients will be invited to complete the questionnaires through face-to-face survey. The patients can answer each question and check the most appropriate opinion (a specific score) in their standards, hopes, pleasures, and concerns. Meanwhile, an investigator in each center will be assigned in the office to help the patients, and to check through each completed questionnaire to ensure that the patients answer all the questions.

##### Economic evaluation

The Cost-Effective Analysis (CEA) and Cost Utility Analysis (CUA) will be assessed through calculation the incremental cost-effectiveness ratio and incremental mean cost per quality-adjusted life year [21]. A specific questionnaire is designed to collect self-reported cost-related data by patients. The total costs include direct costs and indirect costs. The direct costs comprise medical costs and non-medical costs. Besides the costs of four herbal formulas and three drugs, the direct medical costs include diagnostic and lab investigations, oxygen therapy, and the average costs of acute exacerbations managed in the outpatient and inpatient settings. The non-medical costs include traveling expenses, accommodation, catering, and transportation costs to the clinic. The indirect costs were calculated using the human capital method, which consists of duration of sick leaves, lost productivity, travel and waiting time, and nursing.

##### Safety

The routine blood, urine, and stool tests, liver and kidney function tests, and an electrocardiogram are performed. Adverse events will be recorded at any time during the treatment period and follow-up period.

The MMRC, 6MWD, CAT will be recorded at baseline (week 0), in the third month (week 13) and sixth month (week 26) during the treatment period, and at the third month (week 39) and sixth month (week 52) during the follow-up period. The lung function will be observed at weeks 0, 26, and 52. Safety will be measured at weeks 0 and 26. The date of economic evaluation will be measured at week 52.

### Quality control

To ensure the quality of this study, the framework of appropriate planning before the trial, adequate oversight and monitoring, and verification will be executed. The standard operating procedures (SOPs) for trial execution will be implemented to ensure the accuracy and integrity of clinical data at each step of the trial, such as identification, registration, and recruitment. Periodic monitoring will be applied by telephone and email. The completion and compliance of paper case report forms (CRFs), the clinical trial procedures, such as compliance with administration and the withdrawal of participants in each center will also be audited.

### Statistical analysis

The intention-to-treat analysis (ITT) and per-protocol analysis (PP) will be adopted. An ITT analysis will be used to analyze the baseline data and clinical evaluation data of the randomized patients in the groups to which they will be randomly assigned, regardless of their adherence with the treatment, and regardless of subsequent withdrawal from treatment or deviation from the protocol. Partially missing data of the clinical evaluation will be carried forward with the principle of the last visit carried forward (LOCF). PP analysis set will be used to analyze the clinical evaluation data of the patients who are considered to be adherent to the protocol with better compliance. All statistical analyses will be undertaken using SAS9.2 (KEY: FQ37-WSB8-7G5C).

**Figure 1:**
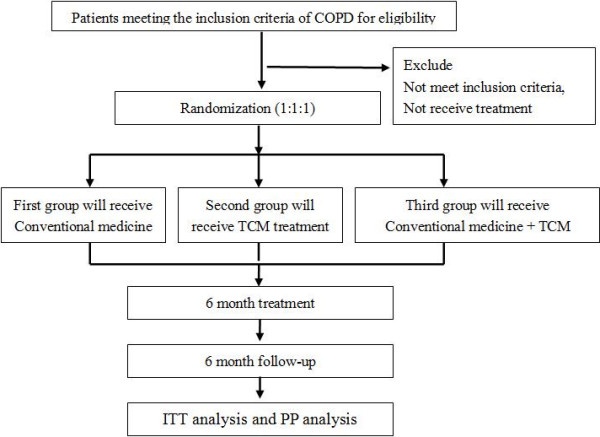
Technical route of this comparative effectiveness research study.

All *P* values will be two-tailed and the α level of significance will be set at 0.05. Measurement data will be presented as mean ± SD, median, inter-quartile range. The one way analysis of variance (ANOVA) will be used firstly to compare the statistical significance among the three groups followed by a post-hoc test such as Tukey’s for multiple comparisons when the data are normally distributed, or a Kruskal Wallis test followed by Mann-Whitney U-tests with a Bonferonni correction will be used if the data are not normally distributed. Since treatment and time course will be investigated, as well as center effect interaction, the analysis of covariance (ANCOVA) will be adopted by using proc GLM for each outcome variable. The repeated measures ANOVA will be used compare the value differences of more than two measures are taken in different time point for the same participants, such as the symptoms, MMRC, 6MWD, CAT before treatment, the third month and sixth month in the treatment period, and the third and sixth months during the follow-up period. The paired-sampled *t* test or signed rank sum test will be used to compare the value differences between pre-treatment and post-treatment within one group. The Chi square test will be used to compare the value differences in safety among the three groups. The cost-effectiveness analysis and cost-utility analysis will be adopted for health economic evaluation. The whole procedure of this study is shown in Figure 1.

## Discussion

Through comparing the applicability and/or superiority of a particular therapy among the existing options in practice or development, CER can narrow the translation gap between clinical research and everyday clinical practice as well as to deliver more cost effective healthcare [22]. It is generally agreed that a wide range of research methods, such as pragmatic clinical trials, prospective observational studies, prospective or retrospective registries, meta-analyses, and technology assessments can be applied in CER [23]. The pragmatic randomized controlled trial (PRCT), with broader inclusion criteria that are more reflective of the general patient population and less strict follow-up monitoring, can achieve a balance between internal validity and relevance, while maintaining the fundamental features of high-quality clinical trials [24,25]. Therefore, the PRCT is adopted by this study to evaluate the effectiveness of three treatments and reflect the experiences of patients in real-world settings and circumstances. To our knowledge, this study will be the first CER in conventional medicine, TCM treatments and a combination of both conventional medicine and TCM treatments in China specifically designed to determine which treatment is the most suitable for COPD patients.

Currently, more and more alternative approaches have been used in COPD patients [26]. Through a series of hard work, the TCM granules, Bu-Fei granule, Bu-Fei Jian-Pi granule, Bu-Fei Yi-Shen granule, and Yi-Qi Zi-Shen granule, have definite effect evidence [13,14,27]. First, based on our long experience in clinical practice, combined with expert counseling many times, the initial framework of the four granules is formed. Meanwhile, through the experimental studies, the mechanism of them is explored and further optimization is performed on its formulas. In addition, the lager sample randomized controlled trials are conducted to evaluate its efficacy. Finally, the four granules are made and used in this study according to strict quality standards.

Due to the nature of pragmatic randomized controlled trial and CER design, the double blinded design is not adopted in our study. Although the study is an open-label trial, however, some measures will be considered to strengthen quality control. To avoid the bias from the researchers’ in the procedure of this study, an investigator separate from all of the clinical researchers will be assigned in each research center as the contact person who preserve and record the randomization information. Therefore, the clinical researchers do not have any effect on enrollment or randomization. Meanwhile outcome assessments will be made by an independent clinical statistician blinded to group allocation and uninvolved in providing intervention or management. However, there are some limitations of the study. On the one hand, the 6-month treatment period and 6-month follow-up duration is a little short to observe the changes in lung function and then show the full effect of three treatments. On the other hand, other therapies for COPD, such as pulmonary rehabilitation, are not set separately. Further studies should be performed to evaluate all COPD treatments.

## Trial status

At the time of manuscript submission, we had recruited 60 patients but not completed the patient recruitment.

## Abbreviations

6MWD: The six-minute walking distance; CAT: COPD assessment test; CER: Comparative effectiveness research; COPD: Chronic obstructive pulmonary disease; FVC: Forced vital capacity; FEV1: Forced expiratory volume in one second; FEV1%: FEV1 percentage of predicted value; GOLD: Global Initiative for Chronic Obstructive Lung Disease; PRCT: Pragmatic randomized controlled trial; TCM: Traditional Chinese medicine.

## Competing interests

The authors declare that they have no competing interests.

## Authors’ contributions

LJS: conception and design, critical revision, and final approval of the manuscript. XY: conception and design, manuscript writing, and final approval of the manuscript. LSY: data collection and analysis, critical revision, and final approval of the manuscript. YXQ: data collection and analysis, and final approval of the manuscript. All authors read and approved the final manuscript.
